# Me and us: Cultivating presence and mental health through choir singing

**DOI:** 10.1111/scs.13078

**Published:** 2022-03-25

**Authors:** Janne Brammer Damsgaard, Svend Brinkmann

**Affiliations:** ^1^ 1006 Health, Department of Public Health, Nursing Aarhus University Aarhus Denmark; ^2^ 1004 Department of Communication and Psychology Aalborg University Aalborg Denmark

**Keywords:** lived experience, mental health, philosophy, psychology

## Abstract

**Background:**

An increasing body of qualitative and quantitative research suggests that choir singing can improve mental and physical health and wellbeing. A recurring phenomenon is social agency and social and emotional competences. However, there is little consensus about the underlying impact mechanisms and the special nature of music as a medium for music‐based social–emotional competence.

**Aim:**

This research was carried out to explore how the participants experienced engaging and singing in the choir *A Song for the Mind* in order to understand the social and emotional aspects in relation to choir singing and mental health.

**Method:**

Six women and two men were interviewed. The study involved open‐ended interviews and applied Paul Ricoeur's phenomenological–hermeneutic theory of interpretation in processing the collected data.

**Findings:**

Two themes emerged—The Singing Me and Cultivating Us. Joining the choir, singing and engaging with the lyrics, helped the participants get in contact with complex feelings and visualise and express challenges. This formed feelings of connecting to oneself and opening up to become aware of the world such as nature, the other person and the choir. Songs, melodies, tones, lyrics—singing together—formed a relation between the participants and the other and the group. This was a meaningful, and to some, a life‐changing experience, and an important learning process to the professionals. As the participants are sensing and connecting to themselves, there is an opening for growing a nascent presence and awareness.

**Conclusion:**

Joining the initiative A Song for the Mind instils an attention to the other person(s). The singing process seems to evoke presence, leading to awareness towards relational aspects and solidarity. In a choir singing perspective, and health care practice in general, this can be seen as a budding and ground‐breaking formation of cultural activities holding learning and empowering potentials instilling mental health.

## BACKGROUND

Since 2000, there has been a considerable growth of interest in singing, wellbeing and health specifically, demonstrating that, for example group singing can have substantial benefits in aiding the recovery of people with a history of serious and enduring mental health problems [1]. Several qualitative and survey studies have also shown significant improvements in affective state after group singing [2, 3, 4]. For example, Bailey and Davidson ( [5] p. 298) present positive effects of participation in group singing in a study that examined interviews and focus groups with 16 members of Canadian choirs from marginalised and middle‐class backgrounds. They proposed benefits of four types: clinical or therapeutic benefits, benefits related to group processes, benefits associated with choir/audience reciprocity and cognitive benefits.

An increasing body of quantitative research likewise suggests that group singing can improve mental and physical health and wellbeing. There are several systematic reviews with attention to specific populations or health outcomes: singing as part of a therapeutic intervention [6], health and older people [7], respiratory function [8], health‐related quality of life outcomes [9] and benefits for people with Parkinson's disease [10]. In addition, the act of singing involves a number of respiratory and neuromuscular processes that may have a relationship with emotion systems, and thereby amplify aesthetic experiences [11]. Clift and colleagues [3] conducted a large (*N* = 1124) cross‐national mixed‐methodological survey assessing choral singers' perceptions of the effects of group singing on samples from choirs in Australia, England and Germany. Of particular interest to the authors were the written comments by participants who disclosed significant challenges in their lives, but still had strong perceptions of the benefits of singing. Examining these responses using a category system based on the WHO definition of health, the authors found repeated self‐reports of physical, psychological and social benefits, and evidence of six mechanisms linking singing with enhanced wellbeing. In support of these results, a systematic review found evidence that participation in ongoing community singing programmes can improve quality of life and social and emotional wellbeing in adults living with chronic conditions [12]. Moreover, an Australian study found that participation in a community choir promoted wellbeing, improved social connectedness, and benefitted health and functioning for a group of adults with mental health and physical conditions [13].

### Social and emotional wellbeing

Music supports social and emotional wellbeing and recovery as documented by Damsgaard and Jensen in an essay exploring how music can support mental health service users' in their recovery process [14]. Scrutinising research literature, 11 key components were identified in which music can support the recovery process. Among the components is social agency, and a recurring phenomenon throughout the components relates to social and emotional competences. Social–emotional competences represent a set of psychological resources, highly relevant for adaptive growth and wellbeing. To Saarikallio, music inherently relates to social–emotional behaviour [15]. For example, evidence shows that musically trained adults outperform musically untrained adults in recognising emotions in spoken sentences. Musical training relates to higher scores in the Trait Emotional Intelligence Questionnaire (TEIQue) and lower scores of alexithymia (using the Toronto Alexithymia Scale [TAS‐20]), that is, the inability to recognise emotions in self and others. Following Saarikallio, evidence is thus emerging about the connections of music engagement to social–emotional competences such as emotion recognition, empathy, pro‐social behaviour and self‐esteem—it being documented that music is conducive to supporting social–emotional skills [15]. However, there is little consensus about the underlying impact mechanisms and the special nature of music as a medium for music‐based social‐emotional competence.

### A Song for the Mind

The present study explored the project *A Song for the Mind*, which is a private‐based choir activity aimed at people with mental health conditions, their relatives and professional facilitators. The initiative is created and founded by the private mental health organisation *SIND Herning Ikast‐ Brande* and the private choir school *Den Jyske Sangskole*. The initiative is a user‐targeted activity promoting meaningful choir lessons. The purpose is to create a recreational offer to people with mental health challenges during or post treatment. The focus is on developing an inclusive community, connecting culture stakeholders with other private and public organisations uncovering meaningful activities, non‐pharmacological ‘treatments’ and un‐explored mental health potentials based on the interaction between the participants and partners. The vision is to create a foundation for developing an arts and culturally based activity to people afflicted with psychological vulnerabilities, independent of age and illness.

## AIM

This research was carried out to explore how the participants (the singers, their relatives and professionals) experienced engaging and singing in the choir *A Song for the Mind* in order to understand the social and emotional aspects in relation to choir singing and mental health.

## METHODS

### Design

Within a phenomenological–hermeneutic approach, a qualitative design was chosen. The study involved open‐ended interviews and applied Paul Ricoeur's phenomenological–hermeneutic theory of interpretation in processing the collected data [16]. This approach was especially suitable because of its focus on narrative interpretation (and narrative understanding) ‘allowing the story to grow out of stories that have never been recounted and that have been repressed in the direction of actual stories …’ [17].

### Participants and data collection

Six women and two men were interviewed and consisted of both mental health service users and professionals—see Table 1. The project leader and the choir leader helped recruit the singers at random amongst persons who were taking part in the activity. The professionals were included because of their profession. Everyone who was asked agreed to participate, and expressed contentment in the opportunity to talk about their experiences with joining and singing in the choir. All interviews took place at Den Jyske Sangskole in a room where we were not disturbed. The data generation was performed by the first author in June 2021. An open‐ended interview guide was used [18]—see Table 2. To achieve openness in the interviews, the participants were given broad and open questions through which they were asked to describe and reflect on how they experienced participating in the choir A Song for the Mind. The researchers' understandings were based on a vast number of studies suggesting more research within the field of users' and professionals' experiences of singing and mental health. Two interview guides were used—one targeting the mental health service users and their relatives, and one targeting the professionals. The interviews commenced with questions such as ‘Can you describe how you experienced participating in *A Song for the Mind*?’ ‘How did you experience the atmosphere within the choir?’ and ‘Can you describe your professional ideas and aims concerning the choir?’ During the interviews, the participants' reflections directed the interviewer, but she specifically aimed to focus on the singers' and professionals' experiences and reflections. Based on this, the interviewer wanted the interviews to be broad and open. The interviews lasted between 35 and 60 min and were subsequently transcribed verbatim.

**TABLE 1:** Data participants

Participant	Gender	Role
A	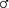	Singer Mental health service user
B	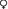	Singer Mental health service user
C	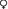	Singer Mental health service user
D	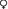	Singer Relative
E	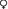	Singer Social worker, facilitator
F	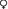	Choir leader, DJS
G	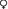	Project leader, DJS
H	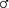	Singer Participant and chairman, SIND

**TABLE 2:** Guides with examples on prepared open questions

**Singers and relatives**
Question: Can you describe how you experienced participating in *A Song for the Mind*?
Question: How did you experience the atmosphere within the choir?
**Professionals**
Question: Can you describe how you experienced participating in A Song for the Mind?
Question: Can you describe your professional ideas and aims concerning the choir?

### Data analysis and interpretation

According to Ricoeur, the aim of a hermeneutic interpretation of a text is to understand the world opened in front of the text [19]. Thus, interpretation means to move from what the text says to what the text speaks about [19]. Striving to interpret, understand and create meaning and thereby achieve deeper insight and new knowledge, the participants' experiences were therefore explored through descriptions gained in the interviews, where the interpretation had already begun. Inspired by Ricoeur's theory of interpretation, we conducted a three‐level interpretation process to reveal the meanings of the participants' experiences [19]. The process included the phases of naïve reading, structural analysis and comprehensive understanding. According to Ricoeur, this method benefits from the dialectical movement between explanation and understanding and provides an understanding of what the text as a whole addresses [19, 20]. In the naïve reading, the text was read several times and with as open a mind as possible to achieve an initial understanding of what it was all about. Ricoeur emphasises that this phase is important, but must be validated by subsequent structural analysis. In the structural analysis, we structured and explained the text by units of meaning (what is said) and units of significance (what the text speaks about)—see Table 3. The analysis was characterised by a dialectical reflection between explanation and interpretation [19]. This allowed us to achieve a deeper understanding of the text, creating themes—see Table 3. The last level of interpretation was conducted as a comprehensive understanding that entailed revising, broadening and deepening the awareness through critical reflection [19]. The themes derived from the texts in the structural analysis became the basis for the comprehensive understanding [19]. Relevant theoretical perspectives and existing research were included to achieve new insight, thus creating new knowledge about the participants' (singers, relatives and professionals) experiences of engaging and singing in the choir.

**TABLE 3:** Example of the analysis process – from quote to theme

Meaning units ‘What is said’	Units of significance ‘What the text speaks about’	Themes
*‘… there is something which can be difficult to put into words … singing can do a lot, it can set you free, and it can make you feel things that you haven't felt before’*.	The text speaks about how choir singing facilitates getting in contact with feelings which prior have been difficult or impossible to sense. This emerging sensation can create an individual understanding and connectedness both to the singer as an individual (self) but also to the other and the group/choir.	The Singing Me
*‘… but it's just a feeling of connection, also within the songs (lyrics), yes – which take you back and which is between us – a connection’*.	The text speaks about how songs/melodies/tones, lyrics and singing create a relation between the self and the other. This is empowering experiences. As the singers are sensing and connecting to themselves, they at the same time see the other person(s) next to them forming a solidary way of being. Such experiences are, within different angles, also met by the professionals.	Cultivating Us

## FINDINGS

### Naïve reading

The naïve reading of the texts showed that the interviewees experienced choir singing as making them get in touch with and capable of expressing emotions, which were otherwise difficult to feel or express. To the group, this induced a strong sense of being present, enabling participants to reach out to each other.

### Structural analysis with themes

#### The Singing Me

Listening to the singers it became clear that they, in different ways, struggled with complex experiences and feelings, which were difficult to express and/or talk about. This could be about challenging or traumatic incidents that created personal challenges affecting everyday life, and dealing with self and others in ways that affect mental health. Participating in the choir was experienced as being ‘set free’, enabling emotions to emerge. One of the singers expressed it like this:

… there is something which can be difficult to give words to … singing can do a lot, it can set you free, and it can make you feel things that you haven't felt before.

The possibility of getting in contact with complex experiences and feelings was described in different ways. One singer explained that joining the choir and singing allowed him to ‘be himself’, enabling him to remove the ‘mask’ he usually put on, hiding existential and psychological struggles, trying to be like others:

I wear a ‘mask’, hiding my struggles. But it is exhausting. Having a place where you don't have to think about that is liberating … having fun, singing, expressing oneself … thinking at a rhythm, a tone, and the voice … it kind of creates, liberates, resources, which makes it easier to be present.

Meeting a safe environment signalling inclusion was important and allowed the singers to ‘un‐mask’, daring to be themselves.

Being present and feeling connected was also instilled by the lyrics of the songs, and this was expressed by several of the singers. One singer talked about how sensations and emotions could be induced by text—picturing nature, following the seasons:

… we followed the seasons … you're using your senses – wauw, this text is actually about how the leaves are fading and then you really notice it. This was also connected to how we could express this vocally … a murmur or a purling sound of the wind. Yes, this sharpens your awareness … to your surroundings.

Another singer expressed how both the song and the lyrics could connect her to others and to important memories reminding her of moving moments in her life:

… it is very valuable to sing. It is something that evokes memories with words that make you recognise … it broadens your horizon, so you are met … in things that you have experienced and been through and yes, have sung with other people.

Singing, being vocal, scrutinising lyrics (being exposed to the flow of nature or other existential aspects) created feelings of being connected ‘backwards’ to one's own history and memories. To the participants, this was an important element in the ability of being able to be present, connecting to themselves, forming their ‘being’—their identity.

This sense of aligning with ‘who you are’, connecting to yourself being present, enhanced the participants' awareness of the group and the other person:

… singing creates resources – when I sing with others I do not have this fear, which I once had, and then I try to make it easier for the others … if I sing aloud, then maybe they will also dare to sing.

To the singers, recognising feelings being connected to oneself was fundamental and a prerequisite of being able to see the other choir members in a deeper way.

#### Cultivating Us

Singing in the choir seemed to illuminate a stance of solidarity and a need for sharing sensations and feelings with the other members of the choir. Telling their stories (one's own story) was important:

But I really yearn for being able to give – giving the others an experience – giving them a feeling, telling a story … and at the same time showing how I feel and how much I feel it …

Singing together with others guided by the choir leader facilitated an opportunity to express and share otherwise hidden feelings. Being able to ‘put into words’—to vocalise—enabled the singers to illustrate and illuminate to other people, for example anger, sorrow, despair or happiness through bodily expressions and through volume and intensity within the voice.

This possibility of being vocal, bodily and being emotional in solidarity, ‘visibility’ and ‘explanation’, was experienced as a huge relief and as being given the opportunity to, without directly using speech/words, materialise, perhaps extreme and unconventional, life occurrences to other people. A singer expressed it like this:

… when you have experienced things and states of mind which other people have not … you can try to pretend that it has not happened, but you can't pretend that you do not feel it.

Singing was seen as ‘another language’ which was easier to align with. This was described in the following way:

… well, I stuttered as a child and found out that if I used another language or sang, it was no longer a problem. There is a lot of good things in singing opposed to talking about things – it takes hours to explain.

Such moments of being able to ‘sing it out’ created feelings of connection to former experiences, to self and to the other choir members:

… but it's just a feeling of connection, also within the songs, yes – which takes you back and which is between us – a connection.

The experiences founded a platform for ‘moving your eyes from the ground and upwards’ – opening up and seeing the choir members, thinking of ways in which to connect and help: *‘… I thought to myself that this could be a possibility for me to help others*’.

Beside recognising oneself and other people, the choir singing also created room for acceptance and being able to contain others, also the occasionally challenging behaviours from some of the choir members:

I try to help as much as I can. But sometimes there can be somebody who REALLY wants to say something – relevant or maybe not so relevant – I accept and do not say – stop it.

This relational and empathic stance was also articulated by some of the professionals:

… you know, I thought to myself: ‘you’re right here, and we have eye contact, and I will be there for you. I can see that you tremble a bit, but that is all right – it is going to be fine.

Such realisations, experiences and interactions were ‘understandings of solidarity’ that run through all the interviews. In different ways, the singers and the professionals all described how singing made people recognise and be open to the other person and the group.

Although the creation of the choir stood on new and innovatory grounds, the professionals had, of course, both theory and practice to stand on. However, even if these ideas and experiences came from other, more ‘traditional’, settings, as activities progressed it became evident that the knowledge of individual and group dynamics within a choir were, to some extent, the same whether you are marked by mental health challenges or not:

… well, we knew that we would focus on ‘self‐management’. We tried to adjust and not to challenge too much. However, in the beginning we put the bar too low – we were afraid to challenge – ‘uh be careful – it is vulnerable people’ – however we learned that ‐ yes, maybe people are vulnerable, yes … until they enter the choir.

To the professionals the singers were vulnerable and marked by complex and different challenges that affected mental health. However, throughout the interviews, it was articulated that the choir singing instilled an awareness of human aspects—cultivating the self and the other.

## COMPREHENSIVE UNDERSTANDING

### The Singing Me

Throughout the interviews, it appears that the singers struggled with complex sensations and emotions having difficulties with expressing and being themselves. Joining the choir, singing and engaging with the lyrics, helped the participants get in contact with complex feelings and visualise and express—somewhat ‘materialise’—for example traumatic and/or social challenges. This formed feelings of connecting to oneself and, simultaneously, opening up to become aware of the world such as nature, the other person and the choir.

To philosopher Martin Buber, in society, there is a tendency to reduce human experience to inner psychological experiences—‘the psychologization of the world’ [21]. This means that we relate to the world as an ‘it’, like a subject to an object [22]. Buber argued that this existential crisis of today is ontological and resulting in a growing (relational) distance between self and the other. He therefore turned to ‘relation’ and ‘dialogue’. To Buber, dialogue does not necessarily require a *spoken* language [22]. Existence and dialogue are related and transcend the limitations of persons holding an ‘inner’ (psychological) space. Buber saw language as a means to express feelings. However, language, as we usually understand it, is not necessarily sufficient to bring the world (i.e. meaningful existence) closer to us ([23] p. 106). To Buber language—‘genuine dialogue’—contains supra‐ or sub‐linguistic ways of expression such as gestures, facial expressions and even silence. The characteristics of a dialogical person are, therefore, the possibility of and ability to ‘be present’. An awareness towards what ‘is’ present, in a movement toward the other, without forgetting oneself. This (presence) is the key to genuine dialogue—to a meaningful life.

Based on these perspectives. it is understandable why the interviewees find relief and comfort in singing. Singing can forward genuine dialogue, connectedness, presence, awareness—meaning in life.

### Cultivating Us

When reading through the interviews it is clear how songs/melodies/tones, lyrics—singing together—form a relation between the participants and the other and the group. This is a meaningful, and to some, a life‐changing, experience, and an important learning process to the professionals. As the participants are sensing and connecting to themselves, there is an opening for growing a nascent awareness.

Buber argued that ‘With(in) the meeting I become myself’ ([23] p. 116). However, to Buber, modern life has reached a critical point. Only through a new interpretation of life in a relation with the other can genuine dialogue and solidarity be restored and the world ‘free itself’ from its de‐humanising crisis. Following Buber, to change the way people live and relate, our language must change. Words like ‘I’, ‘You’ and ‘It’ express and represent a separated and fragmented approach to the world. Therefore, if life is characterised in such ‘atomising’ language, objectifying life, we distort our existence. To Buber the antidote to an objectified, distorted existence is based on the understanding of a link between the way we see and understand ourselves and the way we relate to others [23]. Within this relational perspective, Buber tried to transcend the limitations of personal inner (isolated) experiences, moving towards interhuman relations. To Buber, the word ‘I ‐Thou’, establishes a world of relation and meaning [21] and is characterised by openness, directness, mutuality and presence ([22] p. xii). It is only within this ‘relation’ that personality and the personal really exists. It is about ‘making the other present’, trying to imagine, quite concretely, what the other person is wishing, feeling and perceiving. However, if such ‘education’ shall serve as an effective humanising power in society, Buber argued, then we must cultivate solidarity—the fundamental instinct to relate to the other person—and this is something that begins with the ability of being present.

It appears that joining the initiative A Song for the Mind, does, in different ways, instil an attention to the other person(s). The singing process seems to evoke presence, leading to an awareness towards relational aspects and solidarity. In a choir singing perspective, this focus can forward a culture and ‘educative motion’ towards solidarity and a humanising formation of presence and awareness—between mental health and choir singing.

## DISCUSSION

### Discussion of research

In the study, the participants described that they struggled with complex experiences and feelings, which were difficult to express and/or talk about. This could be about traumatic incidents creating personal challenges affecting everyday life and mental health. Joining the choir was experienced as being ‘set free’, enabling emotions to emerge. The practice of singing together created a vocal and emotional ‘visibility’, which was experienced as a huge relief and as being given the opportunity to, without directly using speech/words, depict perhaps extreme or crucial life occurrences to other people. Singing also furthered the participants to be concerned with the other person. The singing process evoked presence, leading to an awareness towards relational aspects and solidarity.

A qualitative study by Ørjasæter described how music made participants focus on the present moment [24]. It was valued as an empowering experience in everyday life creating ‘flow experiences’ within a world that otherwise seemed to fade away. This created small, positive moments (‘guiding stars’) with potential to change how the participants understood themselves, and thereby allowing them to see their lives in another way as an aid to improved mental health. This supports our findings of being ‘set free’ by joining the choir singing and relating to lyrics, experiencing feelings of being connected to nature, to oneself or the other person. Also, in line with our study, understanding oneself, and the potential role of singing/music therapy as a recovery‐oriented practice, was a key finding in McCaffrey's qualitative study with service users in an inpatient hospital setting [25]. Participants entered an environment where they could ‘really be themselves’ ([25] p. 130) promoting a positive self‐image that built on feelings of achievements and success from participation.

A health‐promoting environment was also described in another study by Ørjasæter [26]. Interacting with art professionals who would see potential instead of limitations helped the participants access opportunities. During the activity, the participants' challenging problems minimised, as they did not have to hide their symptoms or use so much of their cognitive capacity on the effects of the symptoms. ‘In a creative setting there might be more tolerance of diversity, and the idea of “madness” is more acceptable than in traditional health settings’ (p. 1609). This is in line with our results describing how joining the choir and singing allows a singer to ‘be himself’, enabling to remove the ‘mask’ that normally hides existential and psychological struggles in trying to be like others. Similarly, based on a qualitative study, Lloyd and colleagues argue that participation in social and cultural activities can promote (social and emotional) competences and a lifestyle where participants can identify themselves aside from their mental health challenges [27].

Social and emotional competences are also described in a study by Saarikallio [15]. In the study, it is argued that music supports social–emotional skills. Based on substantial evidence that shows how music relates to social–emotional behaviour, theory of music‐based social–emotional competence is presented, proposing a model of *access‐awareness‐agency* defining an interplay of embodied access, reflective awareness and sense of agency. These three components are defined as the core competences that music (in particular) facilitates; competences that underlie and explain further competence in behaviours ranging from affective self‐regulation to social interaction. Although the study targeted ‘music’, this supports our finding that the singing process evokes presence, leading to an awareness towards relational aspects and solidarity. In a choir singing perspective, dialogue/relation between person and person/between Me and Us, can forward competences cultivating agency and a culture comprising solidary (social) and humanising existence, enabling mental health.

### Methodological considerations

This study was based on the interviews of eight users, which is a small number of participants. But since the aim of qualitative studies is to gain a deeper insight into the lifeworld of users, it is not necessarily relevant to discuss the number of participants [28]. It is, however, relevant whether the selected participants can redeem the problem under study [29, 30]. The participants in our study were chosen because of the attachment to the choir A Song for the Mind. Retrospectively, we assessed that the participants were well suited to enter the study, as their stories portrayed that they had reflected on and thought a great deal about participating in choir singing. The phenomenological–hermeneutic approach made it possible for the researchers to establish a close relationship with the patients, which allowed for access to more in‐depth data. Accordingly, initial impressions opened the researcher's mind up to the lived experience and allowed further investigation and interpretation—in a movement between understanding and new exploration. However, to improve trustwortniness, rigour and robustness in a future study, it could be beneficial to include participant observations that focus on the more ‘invisible’ group dynamics.

Still, we entertain the hope that the themes that materialised from the interviews, and the interpretations that evolved, will be of national and international inspiration to mental health users, and to professionals involved in developing activities such as the concept A Song for the Mind, being well aware that the course of each user and professional will always be unique.

## CONCLUSION AND IMPLICATION FOR PRACTICE

This study has shown that singing in a choir—joining the activity A Song for the Mind—can instil meaningful life experiences in the singers. The singers experience that choir singing enables them to get in touch with, and capable of expressing, emotions that are otherwise difficult to express. Joining the choir, singing and engaging with the lyrics, helps the participants get in contact with complex feelings and visualise and express—somewhat ‘materialise’—traumatic and/or social challenges. This gives rise to feelings of being connected to oneself, and, simultaneously, opening up to become aware of the world of nature, the other person, and the choir. Despite struggling with mental health challenges, songs, tones and lyrics create an opening for connection—a relation—between the participants—the other and the group. Joining the choir was experienced as being ‘set free’, getting in contact with complex experiences, enabling emotions to emerge. Singing created a vocal and emotional and social ‘visibility’, which was experienced as a huge relief and as being given the opportunity to, without directly using speech/words, concretise perhaps extreme and unconventional life occurrences to other people. To some of the singers, this is a life‐changing experiences and a crucial learning process to the professionals. As the participants are sensing and connecting to themselves, there is an opening for growing a nascent awareness. Joining the initiative, A Song for the Mind, seems to evoke presence, leading to an awareness towards relational aspects and solidarity. Singing—music—function as a transformative power in reconfiguring a person's experience and behaviour as, it so poetically has been formulated by researchers, a ‘magic mirror’, a ‘beyond the head resource’ or a ‘companion’. In a choir singing perspective and health care practice in general, this can be seen as a budding and ground‐breaking formation of cultural activities holding learning and empowering potentials instilling mental health. Within a mental health care context, choir singing is worth implementing as a dialogical activity that allows individuals to extend their current competence to discover new expanded competence. The social–emotional competence is a set of psychological resources, highly relevant for adaptive growth and wellbeing. Music has been argued to support social–emotional skills, yet there is little theoretical consensus about the underlying impact mechanisms and the special nature of music as a medium for social–emotional competence. Further studies are needed that explore different groups and contexts nationally and internationally.

## ETHICAL CONSIDERATIONS

All participants were informed both verbally and in writing about the purpose of the project. The participants were told that the research was carried out to explore how the singers, their relatives and professionals experienced engaging and singing in the choir *A Song for the Mind* in order to understand the social and emotional aspects in relation to choir singing and mental health. They were assured that participation was voluntary, that they would be able to withdraw from the project at any time and that all data would be made anonymous (Declaration of Helsinki, 1964). According to Danish law, approval from the Regional Committee for Medical Research was not required because of the non‐biomedical character of the study. Approval from the Danish Data Protection Agency was obtained and their requirements for safe data storage were adhered to.

## CONFLICT OF INTEREST

The authors declare no potential conflicts of interest with respect to the research, authorship and/or publication of this article.

## AUTHOR CONTRIBUTIONS

Each author has made substantial contributions to the conception or design of the work and has approved the submitted version and agrees to be personally accountable for the author’s own contributions and for ensuring that questions related to the accuracy or integrity of any part of the work, even ones in which the author was not personally involved, are appropriately investigated, resolved, and documented in the literature. All authors have read and agreed to the published version of the manuscript.
